# Lymphocyte-Specific Protein 1 Regulates Expression and Stability of Endothelial Nitric Oxide Synthase

**DOI:** 10.3390/biom14010111

**Published:** 2024-01-15

**Authors:** Musstafa Smeir, Paulos Chumala, George S. Katselis, Lixin Liu

**Affiliations:** 1 Department of Anatomy, Physiology and Pharmacology, College of Medicine, University of Saskatchewan, 107 Wiggins Road, Saskatoon, SK S7N 5E5, Canada; mms783@usask.ca; 2 Department of Medicine, Canadian Center for Rural and Agricultural Health, University of Saskatchewan, Saskatoon, SK S7N 2Z4, Canada; pab776@mail.usask.ca (P.C.); george.katselis@usask.ca (G.S.K.)

**Keywords:** endothelial cells, eNOS, LSP1, CRISPR-Cas9 gene editing, adenovirus overexpression, mass spectrometry

## Abstract

Nitric oxide (NO), synthesized by endothelial nitric oxide synthase (eNOS), plays a critical role in blood pressure regulation. Genome-wide association studies have identified genetic susceptibility loci for hypertension in human lymphocyte-specific protein 1 (LSP1) gene. LSP1 is recognized as modulator of leukocyte extravasation, and endothelial permeability, however, the role of LSP1 in regulation of NO signaling within endothelial cells (ECs) remains unknown. The present study investigated the role of LSP1 in the regulation of eNOS expression and activity utilizing human macrovascular ECs in vitro and LSP1 knockout (KO) mice. In ECs, specific CRISPR-Cas9 genomic editing deleted LSP1 and caused downregulation of eNOS expression. LSP1 gain-of-function through adenovirus-mediated gene transfer was associated with enhanced expression of eNOS. Co-immunoprecipitation and confocal fluorescence microscopy revealed that eNOS and LSP1 formed a protein complex under basal conditions in ECs. Furthermore, LSP1 deficiency in mice promoted significant upregulation and instability of eNOS. Utilizing a mass-spectrometry-based bottom-up proteomics approach, we identified novel truncated forms of eNOS in immunoprecipitates from LSP1 KO aortae. Our experimental data suggest an important role of endothelial LSP1 in regulation of eNOS expression and activity within human ECs and murine vascular tissues.

## 1. Introduction

Nitric oxide (NO) is a vital molecule to endothelial homeostasis and cardiovascular functions [1,2]. NO is synthesized from two substrates (L-arginine and molecular oxygen) by one of the three distinctive nitric oxide synthase (NOS) enzyme isoforms. Endothelial NO synthase (eNOS) is a homodimer enzyme consisting of two identical monomers [3,4]. For the enzyme to be catalytically active and generate NO, NOS must attach cofactors and dimerize. It has been suggested that tetrahydrobiopterin (BH4) is a critical cofactor for NOS activity [5]. The availability of BH4 as cofactor for eNOS activity is determined via new synthesis from GTP and recycling of dihydrobiopterin (BH2) to BH4 by dihydrofolate reductase [6]. Normally, NOS activity is “coupled”, implying that the oxidation of L-arginine is coupled to reduction of molecular oxygen. However, when there is diminished bioavailability of BH4, the reduction of molecular oxygen is not coupled to oxidation of L-arginine (NOS uncoupling) and eNOS functions analogously to NADPH oxidase (NOX) and generation of superoxide anions rather than NO will ensue. Shear stress and multiple agonists, such as bradykinin and thapsigargin (TG), invoke eNOS activation and increase in NO release via rise in cytosolic calcium concentrations or induction of eNOS phosphorylation [7,8]. It is well known that eNOS in vascular endothelium is subject to multiple phases of regulation at transcriptional, post-transcriptional, and post-translational levels [9].

Hypertension is a highly prevalent cardiovascular health condition that is estimated to affect 1.56 billion people globally by year 2025 [10]. Hypertension is the strong risk factor for heart diseases, cerebrovascular accidents, and renal failure, which are the leading sources of cardiovascular morbidity and mortality [11,12]. Clinical and experimental studies have indicated a crucial role of eNOS-derived NO in the modulation of systemic blood pressure [13,14]. Moreover, significant alterations in eNOS expression have been demonstrated to be strongly associated with the pathogenesis of hypertension [15,16,17], and deficiency of eNOS-derived NO has been considered a prominent feature of endothelial dysfunction in hypertension [13]. 

Lymphocyte-specific protein 1 (LSP1), also known as leukocyte-specific protein 1, is an F-actin binding and cytoskeleton associated protein [18] and calcium-binding protein [19], that was initially identified in lymphocytes, and many cell subsets of hematopoietic origin, such as neutrophils, monocytes, macrophages, dendritic cells, and Langerhans cells [20]. LSP1 expression has recently been reported in human and murine ECs [21,22]. Abnormal pattern of LSP1 expression has been characterized in a variety of malignant neoplasms [23,24,25,26]. LSP1 is a substrate for mitogen-activated protein kinase (MAPK)-activated protein kinase 2 and protein kinase C (PKC) [27,28]. The implication of LSP1 in diverse biological processes, such as inflammation and malignancy, suggests the major regulatory role of LSP1 in signal transduction. LSP1 in granulocytes is expressed in three cellular compartments (60% in cytosol, 25% in the cytoplasmic side of plasma membrane, and 15% in cytoskeleton) [18]. In contrast to leukocyte LSP1, which is mostly expressed in cytosol, endothelial LSP1 is localized mainly in the nucleus, with a small fraction associated with actin cytoskeleton [21]. The expression of endothelial LSP1 predominantly in the nucleus suggests a potential role in transcription. While the role of leukocyte LSP1 has been extensively studied, there is a scientific gap in understanding the functions of endothelial LSP1, particularly whether endothelial LSP1 is involved in the modulation of NO synthesis and endothelial-dependent vasodilatation. Recent genome-wide association studies have revealed numerous genetic variants that are associated with essential hypertension in humans [29,30]. Among those are variants is human LSP1 locus on chromosome 11. This finding was further supported by the experimental data that adult LSP1-knockout (KO) mice had significantly higher mean arterial blood pressure than their corresponding age-matched wild-type (WT) mice, and that endothelium-NO-dependent vasodilatation was impaired in resistance arteries of LSP1 KO mice (our unpublished observations). Given the profound role of eNOS in pathogenesis of hypertension and the evidence of genetic association between polymorphism in human LSP1 gene and development of hypertension, we hypothesized that deficiency of LSP1 would cause aberrant eNOS expression and activity. In this study, we investigated the role of LSP1 in endothelial homeostasis by examining the influence of LSP1 on endothelial prototype marker eNOS using a human endothelial cell (EC) in vitro model (EA.hy926 cells) and LSP1-KO mice. By combining perturbation of function and gain-of-function approaches, we reveal the evidence that LSP1 enhances eNOS expression, maintains the stability of eNOS, and promotes eNOS-derived NO synthesis. Furthermore, co-immunoprecipitation and confocal microscopy demonstrate that LSP1 binds to eNOS in EA.hy926 cells. Together, our data unveil the regulatory role of LSP1 in eNOS expression and activity and provide a novel insight into the molecular mechanism of endothelial dysfunction in hypertension.

## 2. Materials and Methods

### 2.1. Cell Culture

EA.hy926 cells (immortalized human umbilical vein endothelial cell line cells) were purchased from the American Tissue Culture Collection (ATCC, Rockville, MD, USA); were grown in Dulbecco’s modified Eagle medium (DMEM, Cellgro, VA, USA) supplemented with 10% fetal bovine serum (FBS; Hyclone, UT, USA), 100 U/mL penicillin and 100 μg/mL streptomycin (Hyclone, Logan, UT, USA), and HAT supplement (Invitrogen, Carlsbad, CA, USA); and were maintained in 5% CO_2_ at 37 °C. Cells between passages 2 and 6 were used for experiments. 

### 2.2. Lentiviral Packaging

Two LSP1 single guide RNA (sgRNA) CRISPR-Cas9 all-in-one lentivectors and one scramble sgRNA CRISPR-Cas9 all-in-one lentivector were purchased from Applied Biological Materials (Richmond, BC, Canada). Two oligos were designed to target two distinct sequences of the human *LSP1* gene, referred to as LSP1 sgRNA-1 and LSP1 sgRNA-2. The target sequences used in this study were 5′-TTCGAGTGACCCGGGTGCCG-3′ and 5′-TCCAGTTCAGGGGCCTCCGA-3′. Replication-incompetent lentiviruses were generated in HEK293T cells (at 90% confluency in 10 cm dish) via transient co-transfection of the third-generation lentiviral packaging plasmids (GAG-POL, REV, and VSV-G plasmids) and CRISPR sgRNA Cas9 encoding lentivectors using LentiFectin transfection reagent (all purchased from Applied Biological Materials) as per the manufacturer’s protocol. Lentiviral supernatants were harvested twice every 48 h thereafter and were concentrated using Lenti-X Concentrator (Clonetech, Heidelberg, Germany), and the appropriate lentiviral titer was determined using a Lenti-Gostix kit (Clontech).

### 2.3. Lentiviral Transduction and Western Blotting 

EA.hy926 cells were transduced with lentiviruses encoding for CRISPR sgRNA/Cas9 inserts targeting human LSP1 and scramble control lentivirus at a multiplicity of infection (MOI) of 2 in the presence of 12 μg/mL polybrene infection reagent (EMD Millipore, St. Louis, MI, USA) and 10 mM HEPES (Hyclone). Complete media containing polybrene and lentiviruses was left on the cells until they were ready for subculture. Forty-eight hours post transduction, the cells were split in a 1:3 ratio and were subjected to a second round of lentiviral infection. Then, 5 to 10 days after lentivirus transduction, the cells were harvested by cell scraper and lysed in RIPA buffer supplemented with protease and phosphatase inhibitors. Equal amounts of protein (30 μg) were loaded and resolved on 10% SDS PAGE gels followed by semi-dry transfer to nitrocellulose membrane. The membranes were then blocked with 5% skimmed milk in Tris-buffered saline with Tween 20 (TBST) and probed with rabbit polyclonal anti-human LSP1 antibody (1:1000 dilution; Santa Cruz Biotechnology, Dallas, TX, USA, # SC-33160), mouse monoclonal anti-eNOS antibody (1:1000 dilution; BD Biosciences, Franklin Lakes, NJ, USA, # 610297), mouse monoclonal anti-GAPDH (1:2000 dilution; Origene, Rockville, MD, USA, # TA802519), mouse monoclonal anti-β-actin (1:1000 dilution; Santa Cruz Biotechnology, # SC-47778), and rabbit polyclonal anti-GATA-2 antibody (1:1000 dilution; Santa Cruz Biotechnology, # SC-9008).

### 2.4. Adenovirus Packaging and Transduction

Hemagglutinin (HA)-tagged human LSP1 adenovirus (Ad-LSP1) under the control of cytomegalovirus (CMV) promoter and the control CMV-null adenovirus (Ad-CTL) were purchased from Applied Biological Materials. HEK293 cells were cultured in Minimal Essential Medium (MEM; ATCC) supplemented with 10% FBS (Hyclone) and were utilized for adenovirus amplification. Adenoviral particles were harvested when 95% of cells were detached from dishes and were subjected to three freezing/thawing cycles followed by centrifugation at 1500× *g* for 10 min at room temperature to pellet cell debris. For adenovirus transduction, EA.hy926 cells were seeded at 60% confluency one day prior to transduction in 100-mm cell culture dishes and were infected with 5 mL of LSP1 HA and CMV null adenovirus supernatants for 24 h. The protein expression of human LSP1 and eNOS in endothelial cell lysates was determined at 48 h post adenovirus transduction by immunoblotting.

### 2.5. Co-Immunoprecipitation 

For co-immunoprecipitation of native proteins, EA.hy926 cells were grown in 100-mm dishes until they reached confluence, washed twice with ice-cold phosphate-buffered saline (PBS), and were scraped and lysed in CHAPS immunoprecipitation buffer (FivePhoton Biochemicals, San Diego, CA, USA) supplemented with protease and phosphatase inhibitors. Protein lysates were transferred to 1.5-mL Eppendorf tubes, were held for 30 min on ice, and were mixed via gentle repeated inverting and pipetting until samples were homogenous. Insoluble material was cleared by centrifugation at 12,000× *g* for 10 min at 4 °C. The fraction of 3% whole cellular lysate was utilized as input, while the remaining lysate was used for co-immunoprecipitation. Equal amounts of cleared protein extracts were incubated with 8 μg of either anti-eNOS rabbit polyclonal antibody (Abnova Corporation, Taipei, Taiwan; # PAB12680), anti-LSP1 MaxPab rabbit polyclonal antibody (Abnova Corporation; # H00004046-D01), or non-immune rabbit IgG (Cell Signaling, Danvers, MA, USA; # 2729S) overnight at 4 °C with constant rotation. Thereafter, 100 μL of prewashed PureProteome protein A/G magnetic beads (EMD Millipore) were added to immobilize antigen–antibody complexes and were incubated for 1 h at room temperature with end-to-end rotation. The beads were immobilized with a magnetic stand and washed three times in 1 mL of PBST, and protein complexes were released from beads through denaturing elution by heating in 2× NuPAGE LDS sample buffer (Invitrogen) with 100 mM DTT at 95 °C for 10 min. Next, eluted protein complexes along with 3% fraction of input lysate and unbound supernatant (post-IP) were resolved on 10% SDS-PAGE gel, transferred to immobilon-E PVDF membranes (EMD Millipore), and probed with mouse anti-eNOS monoclonal antibody and rabbit anti-LSP1 polyclonal antibody (Santa Cruz Biotechnology). For minimizing the detection of denatured immunoglobulin heavy chain, tidy blot specific horseradish peroxidase HRP-conjugated secondary antibody (Bio-Rad, Hercules, CA, USA) at 1:200 dilution was utilized instead of conventional secondary HRP-conjugated anti-rabbit antibodies. For co-immunoprecipitation of exogenous LSP1, mammalian expression construct coding for human LSP1 tagged with turbo-GFP (tGFP) at the C-terminus was transiently transfected into EA.hy926 cells seeded in 100 mm cell culture dish using Xfect transfection reagent (Clonetech, Mountain View, CA, USA). Seventy-two hours post-transfection, stably transfected cells were selected in the presence of antibiotic G418 at a concentration of 500 μg/mL (Invivogen, San Diego, CA, USA). G418 resistance was conferred by the presence of neomycin resistance gene within the vector. Neomycin resistant colonies were expanded and maintained in DMEM with 10% FBS and characterized for the expression of tGFP-tagged LSP1 protein by immunoblotting. For tGFP-LSP1 co-immunoprecipitation, stably transfected cells were grown in 100-mm cell culture dishes until confluence, washed twice with ice-cold PBS, scraped, and solubilized in NP40-based non-denaturing lysis buffer (Pierce IP buffer, ThermoFisher Scientific, Waltham, MA, USA) supplemented with protease and phosphatase inhibitors. After 30 min incubation on ice with intermittent pipetting, crude cell lysates were centrifuged at 12,000× *g* for 10 min at 4 °C. Protein supernatant was collected and incubated overnight with 10 μg anti-tGFP antibody (Origene; # TA150041) at 4 °C. On the following day, 50 μL of Dynabeads Protein G (ThermoFisher Scientific) were added to antibody protein mixture and incubated for 30 min with constant rotation at room temperature to capture immune complexes. Then, Dynabeads were washed three times with PBST to eliminate non-specific binding, and protein complexes were eluted by incubation in 2× denaturing SDS Laemmli buffer (Bio-Rad) with 100 mM DTT at 95 °C for 10 min. The immunoprecipitated elutes and 3% fraction of input and unbound supernatant were resolved on 10% SDS-PAGE gels.

### 2.6. Confocal Immunofluorescence Microscopy

EA.hy926 cells were plated in 8-well chambered Poly-D-Lysine cell culture slides (BD Falcon, Franklin Lakes, NJ, USA). The media was rinsed with ice-cold PBS and the cells were fixed in 4% paraformaldehyde in PBS. Fixed cells were permeabilized in 0.3% Triton X-100 for 5 min, washed twice for 5 min each with PBS, and then blocked with 8% bovine serum albumin (BSA) for 30 min at room temperature. The cells were incubated with rabbit anti-LSP1 polyclonal and mouse monoclonal anti-eNOS primary antibodies in 1% BSA at 4 °C for overnight, followed by incubation for 2 h at room temperature with Alexa Fluor 647-labelled donkey anti-rabbit and Alex Fluor 488-labelled goat anti-mouse secondary fluorescent probes (Invitrogen; ThermoFisher Scientific), respectively. For colocalization of exogenous LSP1 with eNOS, immunofluorescence staining was performed in stably transfected EA.hy926 ECs expressing human LSP tGFP fusion protein by the incubation of cells with mouse anti-eNOS monoclonal antibody only and followed by detection with Alexa Fluor 568-labelled donkey anti-mouse secondary antibody. The glass slides were mounted with ProLong Gold antifade reagent with DAPI (Invitrogen), and cells were visualized with Zeiss LSM700 inverted confocal microscopy system.

### 2.7. Measurement of Intracellular NO Levels 

NO synthesis was measured by fluorescent staining with DAF-FM diacetate (cell-permeable nitric oxide sensitive indicator; ThermoFisher Scientific) at working concentration of 5 µM in EA.hy926 cells. The cells were incubated with fluorescent probe and the stimulus, thapsigargin, at final concentration of 100 nM in Krebs buffer for 1 h in 5% CO_2_ incubator at 37 °C followed by washing twice with PBS to remove excess fluorescent probe. The fluorescent images were acquired under excitation/emission wavelength (495/515) by Bio-Rad Zoe fluorescent inverted cell imager. DAF-FM mean fluorescence intensities were analyzed using ImageJ (1.52a) software, and statistical analysis was performed using GraphPad Prism (version 5).

### 2.8. Determination of eNOS Dimerization Status

Fresh aortas from the wild-type (WT, 129/SvJ) mice and LSP1-deficient (*Lsp1^−/−^* or KO) mice (14–16 weeks-old) were homogenized separately by cryogrinding in Pierce IP lysis buffer (mild non-denaturing NP40-based lysis solution). Protein extracts were not subjected to boiling step and were mixed with 4× Laemmli sample buffer without reducing agent on ice. The protein samples were loaded on 7% SDS gel and separated using low-temperature SDS-PAGE. Gels and running buffers were equilibrated to 4 °C before electrophoresis and the temperature was maintained at 4 °C by placing Gel tank in ice during electrophoresis. eNOS dimers and monomers were detected by incubating blots with mouse monoclonal anti-eNOS antibody (BD Bioscience).

### 2.9. Experimental Animal Studies

Twelve-to-sixteen-week-old male 129/SvJ (the WT control) mice and *Lsp1^−/−^* mice were used in our studies. The WT 129/SvJ mice were originally purchased from Jackson laboratories, and *Lsp1^−/−^* (KO) mice were generated on 129/SvJ background by homologous recombination as described previously [31]. All animal protocols were approved by the University Committee on Animal Care and Supply (UCACS) of the University of Saskatchewan and met the standards of the Canadian Council on Animal Care. The mice were anesthetized with 60 mg/kg per body weight intraperitoneal pentobarbital and euthanized. The whole aortae and mesenteric arteries were carefully removed and dissected free from perivascular fat in ice-cold DMEM under dissecting microscope and were immediately frozen in liquid nitrogen and stored at −80 °C until use. For the study of eNOS expression, aortae and mesenteric arteries from both the WT and *Lsp1^−/−^* (KO) mice were homogenized by cryogrinding in ice-cold RIPA buffer supplemented with protease and phosphatase inhibitors. The tissue homogenates were cleared from insoluble material via centrifugation at 17,000× *g* for 20 min at 4 °C. The clarified protein extracts were analyzed through immunoblotting for the detection of eNOS and immunoblots were probed with mouse monoclonal anti-eNOS antibodies against the C-terminus of the enzyme (BD Bioscience), mouse monoclonal anti-β-actin antibody (Santa Cruz), and rabbit polyclonal anti-eNOS antibody (Abnova, Taipei, Taiwan; # PAB12680). 

### 2.10. eNOS Immunoprecipitation for Mass Spectrometry

Whole aorta tissue lysates from both the WT and *Lsp1^−/−^* (KO) mice were homogenized separately by cryogrinding in CHAPS-IP lysis buffer with protease and phosphatase inhibitors. After centrifugation (12,000× *g* for 10 min at 4 °C), clarified protein supernatant was incubated (at 4 °C overnight) with 5 μg N-terminus anti-eNOS and 5 μg C-terminus anti-eNOS mouse monoclonal antibodies with constant agitation. Subsequently, eNOS protein complexes were captured with protein A/G magnetic beads as previously described, were eluted and resolved by SDS-PAGE. After electrophoresis, gel was rinsed three times for 5 min each with Milli-Q water to remove SDS. Then, the gel was stained with EZ-Blue staining solution (Sigma-Aldrich, St. Louis, MI, USA) for 24 h at room temperature followed by destaining with water for 2 h at room temperature. Afterwards, bands of interest in the WT and LSP1-KO lanes were excised, in-gel trypsinized, and analyzed via liquid chromatography–tandem mass spectrometry (LC-MS/MS). 

### 2.11. In-Gel Digestion of Proteins

Excised gel bands in eNOS immunoprecipitates from the WT and LSP1-KO aortae, and from CMV-null and LSP1-HA adenovirus-transduced EA.hy926 cells were rehydrated in Milli-Q water, cut into smaller pieces, and destained with 200 mM ammonium bicarbonate (Fisher Scientific, Fair Lawn, NJ, USA) in 50% acetonitrile (Fisher Scientific) at 30 °C for 20 min. The gel samples were dried in Speed-Vac (Labconco, Kansas City, MO, USA) for 15 min and were then submerged in reduction buffer composed of 10 mM dithiothreitol (MP Biomedicals, Solon, OH, USA) in 200 mM ammonium bicarbonate to reduce disulfide bonds. Then, samples were alkylated in 100 μL of 100 mM iodoacetamide (Fisher Scientific) in Milli-Q water in the dark at room temperature for 30 min to prevent reformation of disulfide bonds. The gel pieces were shrunk by adding 100 μL of acetonitrile, the supernatant was removed, and the gel pieces were dried completely in Speed-Vac for 20 min. Next, a digestion buffer consisting of 50 ng/μL trypsin (Pierce, Rockford, IL, USA) in 200 mM ammonium bicarbonate was added in sufficient volume to cover gel pieces and the samples were incubated in a shaker (Eppendorf Thermomixer; Eppendorf, Mississauga, ON, Canada) at 300 RPM at 37 °C overnight. The peptides were extracted in 100 μL of extraction buffer [0.1% trifluoroacetic acid (Fisher Scientific) in 60% acetonitrile] and incubated at 30 °C for 40 min with intermittent vortexing, followed by peptide reconstitution in 0.1% formic acid (Fisher Scientific) to a final concentration of 1 mg/mL for LC-MS/MS analysis.

### 2.12. Liquid Chromatography–Mass Spectrometry (LC-MS/MS)

Extracted peptide samples were analyzed by liquid chromatography–tandem mass spectrometer (LC–MS/MS) in an Agilent 6550 iFunnel Quadrupole time-of-flight (QTOF) mass spectrometer equipped with an Agilent Chip Cube LC-MS interface (Agilent Technologies Canada Ltd., Mississauga, ON, Canada). Chromatographic separation was carried out on a high-capacity Agilent LC Polaris C18 chip. Samples were loaded onto the enrichment column with solvent 0.1% formic acid in water at a flow rate of 2.0 μL/min. Samples loaded to the enrichment column were transferred onto an analytical column, and peptides were separated with a linear gradient solvent system consisting of solvent A (0.1% formic acid in water) and solvent B (0.1% formic acid in acetonitrile), at a flow rate of 0.3 μL/min. The voltages were set at 1900 V (capillary) and at 360 V (ion fragmentor), and drying nitrogen was set at 225 °C with a flow rate of 12 L/min. Positive-ion-mode electrospray ionization MS data were acquired in the mass range of 250–1700 (mass/charge; m/z) for MS1 scan and 100–1700 m/z for MS/MS scans at a scan rate of 8 spectra/s. Data were acquired in data-dependent acquisition mode, and the 20 most intense precursor ions were selected for MS/MS fragmentation. To avoid repeated selection of abundant precursor ions, dynamic exclusion window was set for 0.25 min. 

### 2.13. Peptide Identification and Bioinformatics Analysis

Tandem spectra were analyzed using the Agilent proteomics software package with Spectrum Mill as the protein database search engine. Collision-induced dissociation (CID) fragmentation spectra were extracted and searched against mouse and human species UniProt/Swiss-Prot database. Search criteria included trypsin digestion; maximum of two missed cleavage sites; carbamidomethylation of cysteine as fixed modification; oxidized methionine, carbamylated and acetylated lysine, serine, threonine, tyrosine phosphorylation, asparagine deamidation, N-terminal pyro-GLU of glutamine as variable modifications; precursor mass tolerance 20 ppm; fragment mass tolerance 40 ppm. The retrieved peptide and protein hits were subjected to Spectrum Mill autovalidation at a false discovery rate threshold of <1%.

### 2.14. CRISPR Mismatch Cleavage Assay

CRISPR mismatch cleavage assay and locus-specific PCR based genotyping were utilized to validate sgRNA (CRISPR Genomic Cleavage Detection Kit; Applied Biological Materials). Genomic DNA was harvested from WT and CRISPR-edited cells following the manufacturer’s instructions. Locus-specific PCR was used to yield amplicons flanking sgRNA target sites in LSP1 gene. Heteroduplex formation was conducted by heating PCR amplicons at 95 °C for 10 min in thermal cycler followed by slow cooling to room temperature. Heteroduplex DNA products were digested with T7E1 endonuclease enzyme (New Englands Biolabs, Ipswich, MA, USA) at 37 °C for 1 h. PCR amplicons and digestion DNA fragments were resolved and visualized on 4% precast agarose gel with ethidium bromide (Reliant™ Precast Agarose; Lonza, Kingston, ON, Canada).

### 2.15. Statistical Analysis

All western blotting bands were quantified using Image Lab software (Bio-Rad). Analysis of statistical significance between treatment groups and conditions was performed in GraphPad Prism version 5 software utilizing One-way ANOVA with Bonferroni correction and unpaired Student *t*-test. All data sets were expressed as mean ± SEM. *p* value level < 0.05 was considered statistically significant.

## 3. Results

### 3.1. CRISPR-Cas9-Mediated Genomic Editing of Human LSP1 Gene and Validation of the sgRNAs

**Figure 1:**
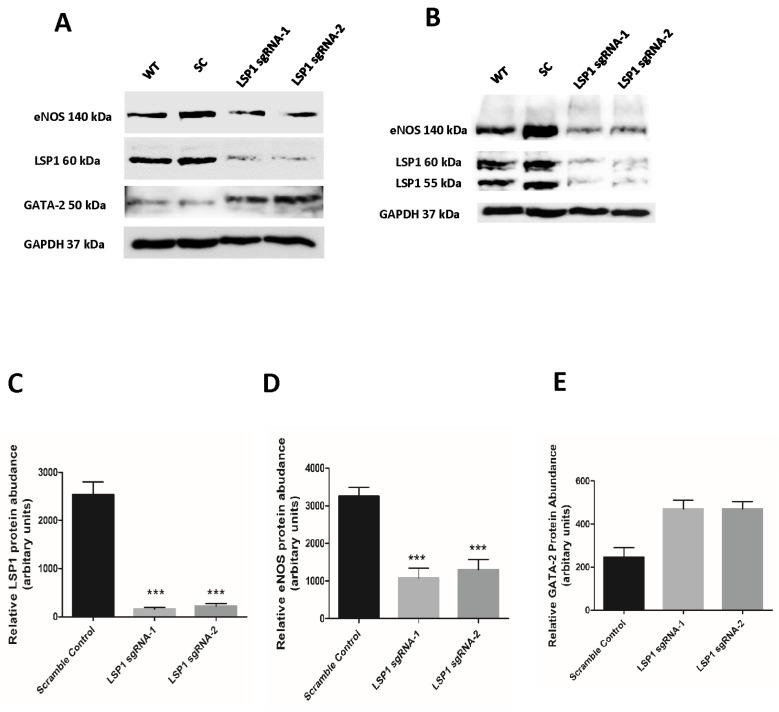
Knockout of human LSP1 gene in EA.hy926 cells by lentivirus delivery of CRISPR sgRNA/Cas9 vectors. Cultured EA.hy926 cells were transduced with sgRNA Cas9 all-in-one lentivriuses against two distinct domains of the human LSP1 gene (sgRNA-1 and sgRNA-2) or scramble control (SC) sgRNA Cas9 all-in-one lentivirus. (**A**) Representative western blots of wild-type (WT), scramble control (SC), and LSP1 sgRNA-1 and sgRNA-2 KO groups probed for human eNOS, LSP1, GATA-2, and GAPDH loading control. (**B**) Protein expression of 55 and 60 kDa LSP1 isoforms by immunoblotting following *Lsp1* knockout induced by CRISPR-Cas9 machinery. Quantitative expression analysis as assessed by immunoblotting for LSP1 (**C**), 140 kDa eNOS (**D**), and GATA-2 (**E**) in CRISPR-edited EA.hy926 ECs. eNOS and LSP1 data are presented as mean ± SEM from at least four independent experiments. GATA-2 data are representative of two independent experiments. Differences were calculated using one-way ANOVA with Bonferroni’s post hoc test. *** denotes *p* value < 0.001.

**Figure 2:**
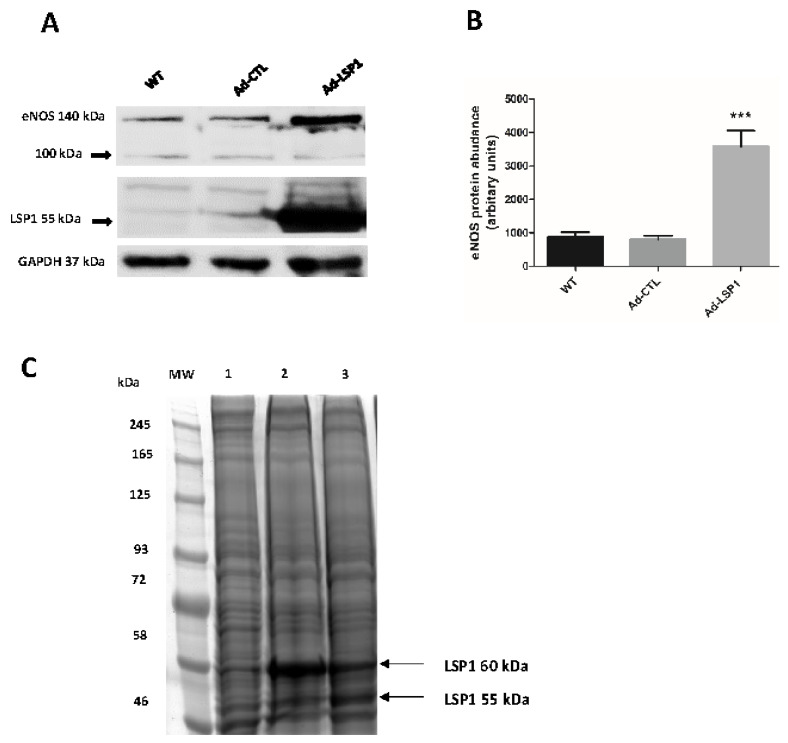
Overexpression of human LSP1 via adenoviruses upregulates the expression of eNOS. EA.hy926 cells were transduced with LSP1-encoding adenovirus (Ad-LSP1) or control CMV null adenovirus (Ad-CTL) for 24 h. (**A**) The protein expression of human LSP1, eNOS, and GAPDH loading was determined at 48 h after adenovirus transduction by immunoblotting. (**B**) Quantitative analysis of eNOS protein expression in untransduced wild-type (WT) control and Ad-CTL and Ad-LSP1 transduction groups. Statistical analysis was performed by one-way ANOVA with Bonferroni’s post-hoc test. *** indicates *p* value < 0.001, *n* = 4. (**C**) Crude cellular lysates from EA.hy926 cells transduced with control CMV-null adenovirus (Lane 1), LSP1 adenovirus (Lane 2 and Lane 3) were resolved on 10% acrylamide gel and stained with colloidal Coomassie blue. Two prominent protein bands at MW of 55 and 60 kDa were excised, in-gel trypsin-digested, and identified as LSP1 isoforms via tandem mass spectrometry.

For the LSP1 KO studies, EA.hy926 cells were transduced with two distinct LSP1 sgRNA CRISPR-Cas9 lentiviral constructs to improve specificity and minimize off-target effects. The resultant KO groups are referred to as LSP1 sgRNA-1 and LSP1 sgRNA-2. We have confirmed successful CRISPR-Cas9 genomic editing of human LSP1 gene by immunoblotting, and we have achieved very significant knockout of human LSP1 (Figure 1A,B). We have validated sgRNA-mediated CRISPR-Cas9 editing via locus-specific PCR-based genotyping and T7E1 endonuclease assay (Figure S1). Two different populations of EA.hy926 cells transduced with LSP1 sgRNA-2 lentiviruses displayed two predicted mismatch cleavage bands at molecular weights of approximately 614 and 377 base pairs (bp), indicating successful CRISPR editing at the LSP1 target locus, whereas the WT control exhibited only a single band at 1011 bp (Figure S1A). Furthermore, we have utilized PCR-based approach to genotype CRISPR edits induced by LSP1 sgRNA-1. Following PCR amplification of sgRNA-1 target locus, we ran PCR amplicons on 4% agarose gels for 1 h. This approach has recently been demonstrated to be efficient for detection of a small insertions and deletions generated by CRISPR-Cas editing machinery [32]. Three mutant transduced cells generated via sgRNA-1 CRISPR-Cas9 showed homozygous mutations, as evidenced by small (Mutant 1 and 3) insertions and large (Mutant 2) deletion (Figure S1C). Attempts to digest PCR amplicons from LSP1 sgRNA1 target sites with T7E1 endonuclease were unsuccessful, suggesting that indels were predominantly homozygous mutations as T7E1 endonuclease typically recognizes and cleaves mismatched DNA in mutant heteroduplexes. In addition, we verified CRISPR-Cas9 editing via observation of protein-level mutation, and we demonstrate that genomic editing mediated by sgRNA-1 CRISPR-Cas9 generated truncated LSP1 proteins via frameshift mutation (Figure S2, lower panel). Our data indicate that the knockout is mostly apparent at the expected molecular weight 60 kDa of human LSP1 as shown in the previous reports [20,28]. However, we have also observed a very significant knockout of LSP1 at a molecular weight of 55 kDa (Figure 1B, middle panel). The LSP1 55 kDa band potentially represents another differentially phosphorylated form of human LSP1, which has been designated as P55 in previous studies [27]. In our study, we have characterized the expression of human LSP1 as two differentially phosphorylated forms in ECs by LC-MS/MS (Figure 2C), and we have validated distinct phosphorylation sites of both 55 kDa and LSP1 60 kDa LSP1 isoforms (Supplementary File S1). Amino acid percent coverages for 60 kDa and 55 kDa LSP1 were 88.7% and 65.7%, respectively. A total of 95 and 13 unique peptides (matched queries) for 60 kDa and 55 kDa LSP1 isoforms, respectively, were confidently identified in our tandem MS analysis. It remains to be fully elucidated what the precise function of each LSP1 isoform in ECs is.

### 3.2. Knockout of Human LSP1 Reduces eNOS Gene Expression in EA.hy926 Cells

To address whether LSP1 is crucial in regulation of eNOS gene expression, we have measured the expression of eNOS at protein levels in LSP1 KO cells and observed substantial reduction in eNOS expression, i.e., more than two-fold downregulation of eNOS protein expression for both sgRNA-1 and sgRNA-2 LSP1 KO groups vs. that for the scramble controls (Figure 1). Furthermore, our data suggest that perturbation of LSP1 via CRISPR-Cas9 markedly upregulated GATA-2 expression (Figure 1A). Surprisingly, we have detected truncated LSP1 protein bands in one population of LSP1 sgRNA-1 KO cells, suggesting that LSP1 transcripts harboring frameshift mutation produced C-terminal truncated LSP1 protein at high expression levels (Figure S2, lower panel). Exon skipping and formation of truncated proteins following CRISPR-Cas9 editing have been demonstrated in previous studies [33,34]. Truncated LSP1 proteins were identified by probing immunoblot with rabbit anti-human LSP1 C-terminus polyclonal antibody in two separate experiments. The complete absence of bands in both WT and scramble control lanes ruled out the possibility of non-specific bands. Truncated LSP1 proteins did not completely restore the expression of eNOS back to normal levels, suggesting that they might have a dominant negative function or impaired activity compared to the full-length LSP1 protein.

### 3.3. Human LSP1 Overexpression Enhances the Expression of eNOS

To further examine whether endothelial LSP1 has a positive role in regulation of eNOS expression, we tested whether overexpression of human LSP1 would promote eNOS expression. As shown in (Figure 2, middle panel), we have obtained robust expression of human LSP1 at 48 h following adenoviral transduction. Our results indicate that overexpression of human LSP1 via adenoviruses markedly upregulates the expression of eNOS (more than three-fold upregulation) compared to CMV-null adenovirus control (Figure 2A, upper panel). Collectively, data in Figure 2 strongly suggest that LSP1 is a positive regulator of eNOS expression. 

### 3.4. LSP1 and eNOS Interact Physically in Human Macrovascular Endothelial Cells

**Figure 3:**
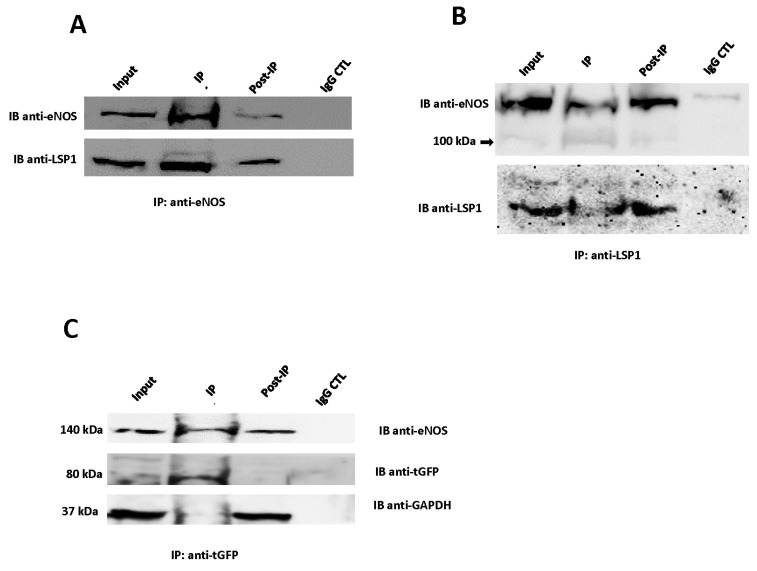
eNOS and LSP1 interaction in human EA.hy926 endothelial cells. (**A**) Representative immunoblotting of eNOS immunoprecipitate. EA.hy926 cells were lysed in CHAPS IP lysis buffer and clarified protein extract was incubated with anti-eNOS rabbit polyclonal antibody or isotype control IgG. Immunoprecipitated proteins were subjected to western blotting analysis with mouse anti-eNOS monoclonal antibody (upper blot) and rabbit anti-LSP1 polyclonal antibody (lower blot). Data are representative of at least four independent experiments. (**B**) Reverse co-immunoprecipitation reveals the association of LSP1 with wild-type (WT) 140 kDa eNOS and truncated 100 kDa eNOS. Data are representative of at least two independent experiments. (**C**) Co-immunoprecipitation of LSP1-tGFP and eNOS. EA.hy926 cells stably transfected with tGFP-LSP1 construct were lysed in Pierce IP lysis buffer and purified protein extracts were subjected to immunoprecipitation by anti-tGFP mouse monoclonal antibody or non-immune mouse IgG. Fractions (input, IP elute, and post-IP supernatant) were resolved by SDS-PAGE electrophoresis and labelled with anti-eNOS mouse monoclonal antibody (top), and mouse anti-turbo GFP monoclonal antibody (middle), and anti-GAPDH mouse monoclonal antibody (bottom). Blots representative of three experiments are shown.

To decipher whether human LSP1 interacts physically with eNOS in ECs, we performed eNOS immunoprecipitation in EA.hy926 cells under non-denaturing conditions. As shown in Figure 3A (lower panel), LSP1 was specifically immunoprecipitated in the elute fraction, suggesting that eNOS and LSP1 were associated with each other as protein complex in ECs. As seen in the lane of post-IP fraction (Figure 3A), eNOS band was faint, implying successful immunoprecipitation of majority of eNOS protein complexes. No LSP1 signal was observed in the control lane, where IP antibody was substituted with normal rabbit IgG indicating the specificity of the interaction between eNOS and LSP1. To further support our hypothesis that LSP1 binds to eNOS in human ECs, we performed reciprocal immunoprecipitation of endogenous human LSP1. As observed in Figure 3B, eNOS was detected in LSP1 immunoprecipitate, further supporting our findings that LSP1 and eNOS form a protein complex in ECs. To provide additional evidence of LSP1 interaction with eNOS, we have transfected human LSP1-turboGFP cDNA construct into EA.hy926 cells and we have generated stable EC line expressing tGFP-LSP1 fusion protein. Immunoprecipitation of tGFP revealed the association of tGFP-LSP1 and eNOS (Figure 3C, upper panel). To demonstrate the specificity of the interaction, we substituted anti-tGFP antibody for isotype matched control IgG and we probed immunoblots with anti-GAPDH antibody, a protein not expected to interact with LSP1 (Figure 3C, lower panel). 

**Figure 4:**
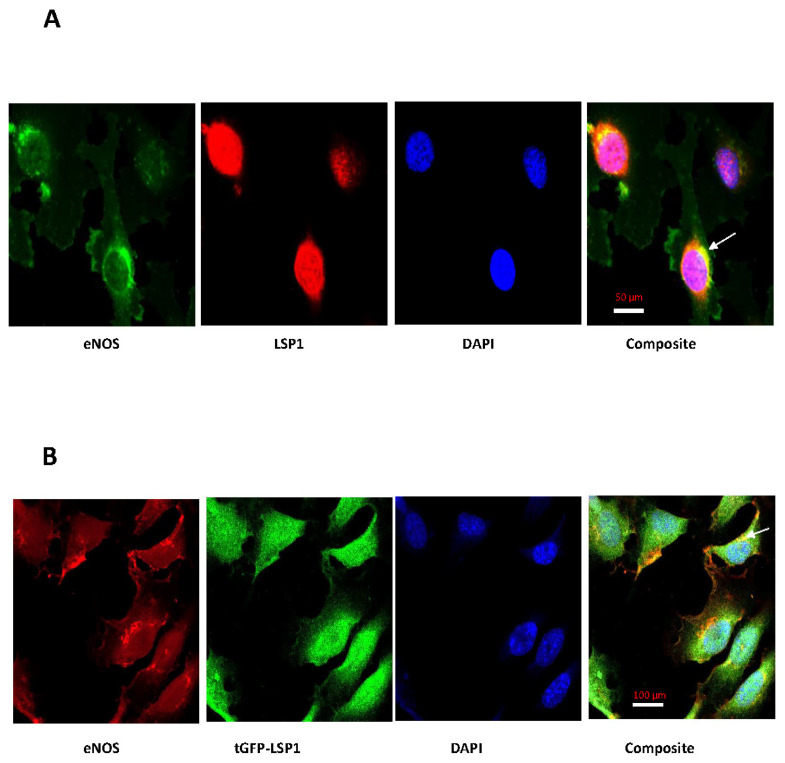
(**A**) Colocalization of native LSP1 and eNOS in human macrovascular ECs. EA.hy926 cells were cultured on poly D-lysine treated tissue culture glass slides and were fixed, permeabilized, and immunolabelled with mouse monoclonal anti-eNOS (green) and rabbit polyclonal anti-LSP1 (red) primary antibodies, followed by detection with Alex-Fluor-488-labelled goat anti-mouse and Alexa-Fluor-647-labelled donkey anti-rabbit secondary fluorescent probes. Note the typical localization of eNOS to perinuclear region and distinct plasma membrane pools, with fainter staining in cytosol. Arrow denotes significant colocalization (yellow) of eNOS and LSP1 in the composite image, particularly in perinuclear region. Immunofluorescence images were captured by confocal microscope using 63× oil lens. (**B**) Colocalization of exogenous LSP1-tGFP and eNOS in human macrovascular ECs. EA.hy926 cells stably transfected with LSP1-tGFP construct were exclusively immunolabelled with mouse monoclonal antibody to eNOS (red), followed by identification with Alexa-Fluor-568-conjugated donkey anti-mouse secondary antibody. Arrows indicate areas of colocalization of the two proteins in the composite image (yellow). Turbo-GFP tagged human LSP1 colocalizes with eNOS in distinct perinuclear distribution. The pattern of colocalization of exogenous LSP1 is consistent with native LSP1 colocalization with eNOS. The blue fluorescence represents EC nucleus staining with DAPI. The images were acquired by Zeiss LSM700 inverted confocal microscope using 40× oil lens.

To gain insight into subcellular localization of human LSP1 interaction with eNOS in ECs, we characterized the distribution of LSP1-eNOS protein complexes in EA.hy926 cells by confocal microscopy utilizing anti-eNOS and anti-LSP1 antibodies. Both human LSP1 and eNOS were expressed largely within their anticipated subcellular compartments in ECs. As observed in Figure 4A (green), eNOS resides predominantly in perinuclear compartment and in distinct areas of plasma membrane with scattered weak signal in cytosol, whereas native LSP1 was primarily localized in the nucleus and additional signal emanating from perinuclear structures (Figure 4A, red). As seen in the composite image of Figure 4A, human LSP1 colocalized with eNOS in a characteristic semilunar perinuclear distribution pattern (yellow). Next, we investigated the distribution and colocalization of exogenous LSP1-tGFP and eNOS in ECs. To delineate if LSP1-tGFP fusion protein colocalizes with eNOS, ECs stably expressing tGFP-LSP1 were solely immunolabelled with mouse anti-eNOS monoclonal antibody. As seen in the composite image of Figure 4B, tGFP-LSP1 colocalizes with eNOS mostly in perinuclear region (yellow) with discrete areas of colocalization in the plasma membrane. 

### 3.5. Impaired NO Production in LSP1-Deficient Human Endothelial Cells

**Figure 5:**
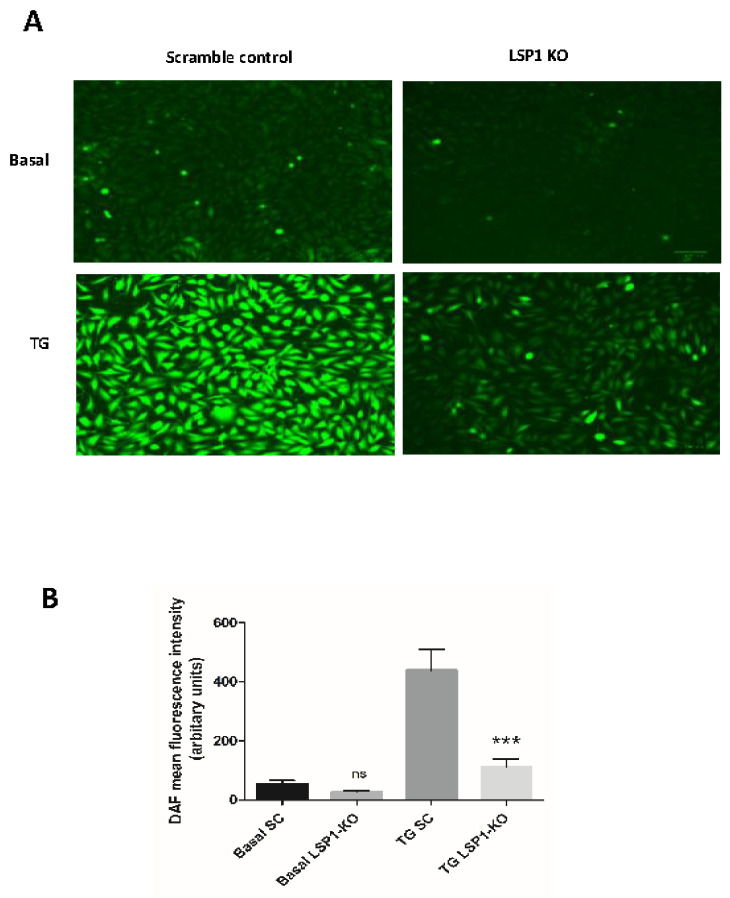
The effect of LSP1 knockout on basal and agonist-induced NO release in human endothelial EA.hy926 cells. Seven days post-transduction with scramble control (SC) or LSP1-sgRNA CRISPR-Cas9 lentiviruses, EA.hy926 cells loaded with DAF-FM were stimulated with thapsigargin (TG, 100 nM) for 1 h. (**A**) Fluorometric quantification of basal and thapsigargin-stimulated NO production in SC and LSP1 KO groups. (**B**) Quantitative analysis of mean fluorescence intensity of the data from (**A**). Quantification of intracellular fluorescence was determined by ImageJ in two wells per treatment group in each of the four experiments. ns denotes not significant; *** denotes *p* value < 0.001 according to one-way ANOVA. Scale bar = 100 μm.

To assess functional consequences of LSP1-eNOS interaction in EA.hy926 cells, we measured intracellular NO production by NO-specific fluorescent probe DAF-FM diacetate following the knockout of EC LSP1. Since eNOS is a calcium/calmodulin-dependent enzyme, we have assessed both basal and TG-stimulated NO release. Thapsigargin has been proven to be the most potent calcium mobilizing agonist for stimulation of NO production in ECs [35]. As shown in Figure 5A,B, there were no significant differences in basal NO output between the scramble control and LSP1-KO groups. However, TG-stimulated NO synthesis was significantly impaired in LSP1-KO ECs compared to scramble control, suggesting that calcium-dependent activation of eNOS is blunted following knockout of human LSP1. 

### 3.6. LSP1 Deficiency Leads to Increased Expression of eNOS with eNOS Instability In Vivo and Enhanced Susceptibility of eNOS to Proteolytic Degradation in LSP1 KO Mice

**Figure 6:**
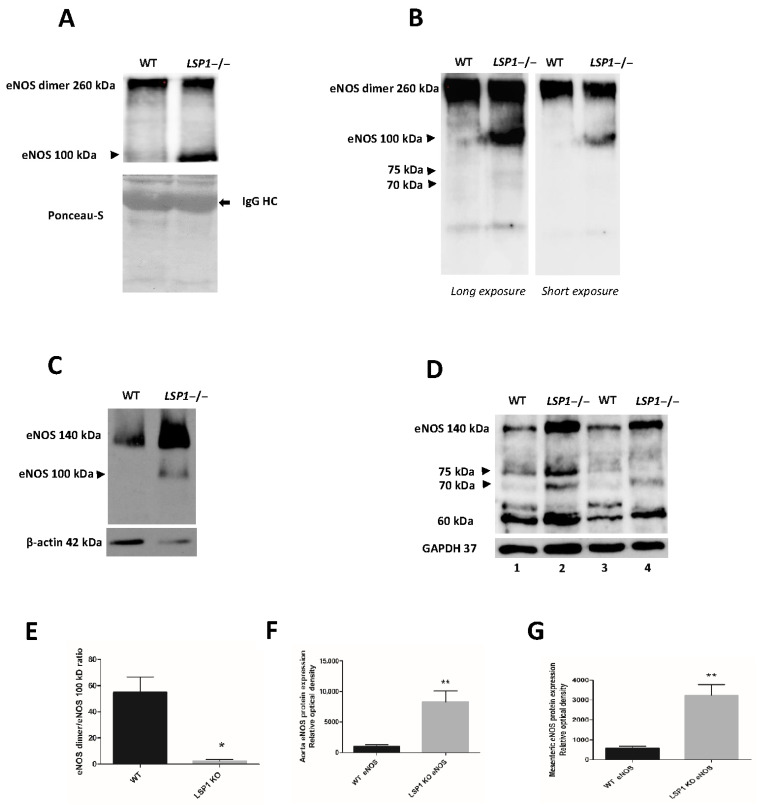
eNOS expression in vascular tissues from WT and LSP KO mice. (**A**) eNOS dimerization and dissociation of eNOS dimers to 100 kDa fragments in vivo in LSP1-KO aortae. eNOS was immunoprecipitated from the WT and LSP1-KO aorta tissue lysates followed by immunoblotting with mouse anti-eNOS monoclonal Ab. The experiment was repeated two times with similar results, *n* = 2. IgG HC is immunoglobulin heavy chain. (**B**) eNOS instability in vivo in LSP1-KO aortae. Original uncropped immunoblot of crude aortic tissue lysates subjected to LT non-denaturing SDS-PAGE, probed with mouse anti-eNOS monoclonal Ab. Prominent 100 kDa, and faint 75 and 70 kDa eNOS immunoreactive bands were only observed in LSP1-KO aorta lane. (**C**) eNOS protein expression in aortae from age-matched WT and LSP1 KO mice. Immunoblots shown are representative of four independent experiments, *n* = 4. (**D**) eNOS cleavage in vascular tissues of LSP1 KO mice. Aortae and mesenteric arteries from WT and LSP1-KO mice were lysed in CHAPS lysis buffer. The immunoblots were probed with rabbit anti-eNOS polyclonal antibody (top), and GAPDH (bottom). The rabbit anti-eNOS polyclonal antibody has revealed 75 kDa and 70 kDa immunoreactive bands which were the cleavage fragments of eNOS. The 75 and 70 kDa bands were recognized as eNOS by LC-MS/MS analysis. Data are representative of three independent experiments. Lane 1 and Lane 2 represent aorta protein samples, whereas Lane 3 and Lane 4 represent mesenteric arterial lysates. Arrowheads in all immunoblots indicate eNOS truncated fragments. Graphs demonstrate the quantification of eNOS dimer-to-eNOS 100 kDa ratio (**E**), and the relative eNOS protein abundance in aortae (**F**) and mesenteric arteries (**G**) of the WT and LSP1-KO mice. The results are displayed as mean ± SEM. * denotes *p* value < 0.05; ** denotes *p* < 0.01 according to one-way ANOVA with Bonferroni’s post hoc test.

**Figure 7:**
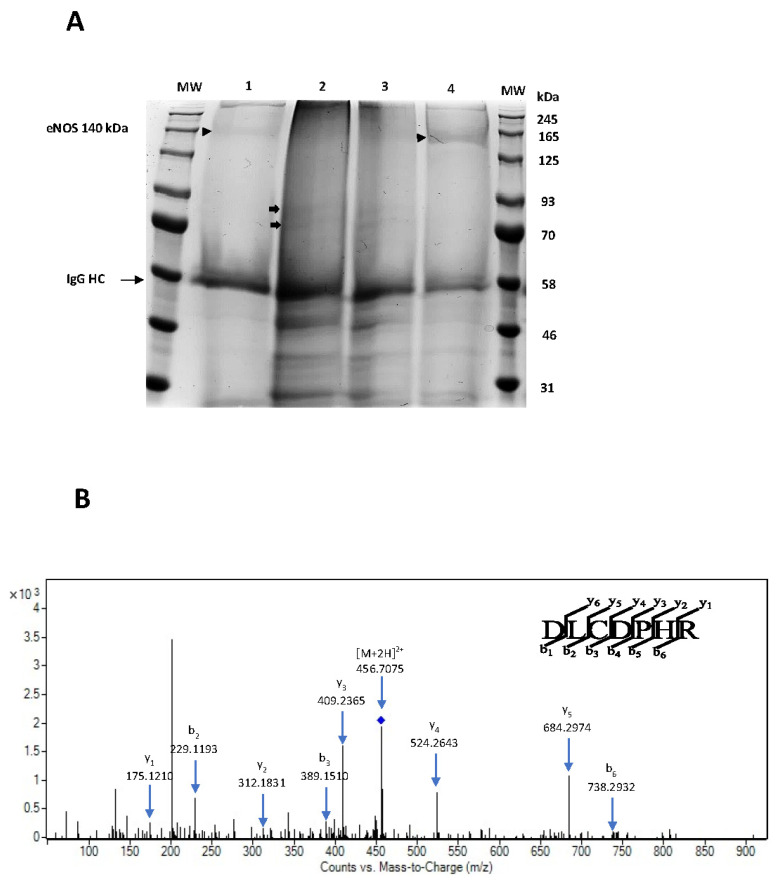
Affinity purification of eNOS for liquid chromatography–tandem mass spectrometry (LC-MS/MS) analysis. (**A**) Tissue protein extracts from WT aortae (Lane 1), LSP1 KO aortae (Lane 2), and from EA.hy926 cells transduced with control CMV-null adenovirus (Ad-CTL, Lane 3) and LSP1-encoding adenovirus (Ad-LSP1, Lane 4) were subjected to immunoprecipitation with anti-eNOS monoclonal antibody. eNOS immunoprecipitates were resolved on 10% acrylamide SDS-PAGE and stained with colloidal Coomassie blue. Two bands, indicated by black arrows at MWs of 75 kDa and 70 kDa, were unique to eNOS immunoprecipitates from LSP1 KO aortae lane and were recognized as eNOS by LC-MS/MS. Arrowheads represent full-length eNOS identified via tandem mass spectrometry. Several novel phosphorylation sites were identified by LC-MS/MS analysis in eNOS band (indicated by arrowheads) purified from LSP1 overexpressing ECs lysate. The ladder lane shows molecular weight markers (245, 165, 125, 93, 70, 58, 46, and 31 kDa). (**B**) QTOF MS/MS spectra generated by CID of a representative eNOS tryptic peptide (DLCDPHR) from in-gel digestion of the 70 kDa band, indicating the b- and y-ion series derived from the doubly charged ion [M + 2H]^2+^ at 456.7 m/z.

Given the complex nature of NO signaling in vivo, we sought to further examine the impact of LSP1 gene knockout on eNOS expression. First, we investigated whether endothelial dysfunction in LSP1-KO mice is associated with eNOS instability, we immunoprecipitated native eNOS from aortae of the WT control mice and LSP1-KO mice followed by low-temperature (LT) SDS-PAGE to study the dimerization of eNOS in vivo. We also performed non-denaturing LT SDS-PAGE to investigate eNOS dimerization and stability in aortae of LSP1-KO mice. As shown in Figure 6A,B, the formation of eNOS dimer at 260 kDa was less in isolated aortae of LSP1 KO mice, associated with prominent appearance of eNOS 100 kDa band, whereas eNOS from aortae of age-matched WT mice showed prominent eNOS dimer band with no detectable 100 kDa eNOS protein. eNOS dimer/eNOS 100 kDa band ratio (Figure 6E) was significantly higher in WT compared to LSP1-KO mice. These data suggest that adequate expression of LSP1 is vital for maintaining dimerization status of eNOS in vivo. Next, we performed immunoblotting to quantify eNOS expression on aortae and mesenteric arterial tissue homogenates from age-matched WT mice and LSP1-KO mice under denaturing conditions. As shown in (Figure 6C,D, upper panels), the expression of eNOS was significantly increased in the aortae and mesenteric arteries of LSP1-KO mice compared to their corresponding age-matched WT mice. eNOS protein abundance increased by more than seven-fold for aortae and more than four-fold for mesenteric arteries (Figure 6F and Figure 6G, respectively). Besides eNOS dysregulated expression and eNOS dissociation/uncoupling in LSP1-KO mice, we have observed truncated eNOS immunoreactive bands in aortae and mesenterial arteries tissue lysates from LSP1-KO mice at molecular weight of 100 kDa (Figure 6C, upper panel), and at molecular weight of 75 kDa and 70 kDa (Figure 6B,D). To gain further insight into the identity of these immunoreactive bands, eNOS protein complexes were affinity-purified from WT and LSP1-KO aortae utilizing mouse anti-eNOS monoclonal Ab. eNOS immunoprecipitates were resolved on SDS-PAGE gel and were stained with colloidal Coomassie blue for 24 h. Subsequent protein analysis by LC-MS/MS confirmed successful immunoprecipitation of mouse eNOS with retrieved amino acid coverage (48%) as well as significant number of unique peptides (60 matched queries) identified for WT eNOS (Figure 7A, Lane 1). Furthermore, two bands at molecular weights of 75 and 70 kDa (Figure 7A, Lane 2) were unique to eNOS immunoprecipitate in LSP1-KO lane, were excised, and in-gel-digested with trypsin. Tryptic digest was analyzed via LC-MS/MS, and tryptic peptides were searched against the Swiss-Prot protein database. A total of seven unique peptides from the 75 kDa band and nine unique peptides from 70 kDa band matched eNOS prototype sequence in the database. Amino acid percent coverage was 6% and 9% for 75 kDa and 70 kDa eNOS, respectively. Figure 7B shows MS/MS spectra of unique eNOS peptide from in-gel digestion of 70 kDa band. We have validated this eNOS peptide (Supplementary File S2). Supplementary Table S1 shows additional unique eNOS peptide 497-516 (EVANAVkISAsLmGTVMAKR) identified in 75 kDa band where lysine is acetylated. Acetylation of lysine residues in the calmodulin binding domain of eNOS has been associated with inhibited eNOS activity and endothelial dysfunction via impeding calmodulin binding [36]. Despite identifying the same peptide in full length eNOS band (EVANAVK) from the immunoprecipitates of the WT mice, lysine residue (K) was not acetylated, suggesting that acetylation in calmodulin binding domain was specific to eNOS proteins in LSP1-KO mice. It has been previously demonstrated that acetylated proteins can be targeted for degradation [37]. We are not sure whether acetylated eNOS in LSP1-KO mice is marked for degradation via a proteasomal pathway. Together, these data support our proposed hypothesis that LSP1 confers eNOS protection against proteolytic degradation or truncation.

## 4. Discussion

Although endothelial LSP1 has been previously studied in the context of inflammation and endothelial permeability, there has been a lack of information regarding the role of LSP1 in endothelial dysfunction, particularly the impact of LSP1 on endothelial hallmark molecule eNOS. In this study, we hypothesized that LSP1 dictates endothelial function via modulation of expression and function of eNOS, and we have tested our hypothesis utilizing an in vitro EC culture model and LSP1 knockout mice. We have chosen EA.hy926 cells as an in vitro model to study the role of human LSP1 in endothelial dysfunction because these cells maintain differentiated endothelial cell phenotype [38], conserve many features and markers from primary HUVEC [39,40], and are presently the best-characterized macrovascular endothelial cells [39].

Although actins and actin-binding proteins (ABPs) were initially considered structural proteins, there have been recent reports advocating that actins and ABPs may act as regulators of transcription [41,42,43]. Furthermore, recent study has identified nuclear β-actin as a novel transcriptional modulator of eNOS expression [44], providing evidence that transcriptional activity of actins and ABPs exist within human EC. Because LSP1 is inherently an ABP [18,45,46] and is predominantly sequestrated in the nucleus of EC [21], it is possible that LSP1 regulates eNOS gene expression at the transcriptional level. To test our hypothesis that LSP1 modulates the expression of eNOS at transcriptional level, we performed immunoblotting to quantify gene expression of eNOS in cultured EA.hy926 cells following depletion of LSP1 by CRISPR-Cas9 system, and we observed significant downregulation of eNOS at protein level. Our results were further supported by the evidence that adenoviral-mediated overexpression of human LSP1 in EA.hy926 cells led to a significant upregulation of eNOS protein expression. Collectively, these data suggest that LSP1 positively regulates the expression of eNOS. Next, we investigated whether GATA-2, a transcription factor that is known to bind to eNOS promoter region and regulates basal eNOS expression [47], is involved in LSP1-mediated regulation of eNOS expression. Our data suggest that LSP1 modulates the expression of eNOS independently of GATA-2 (Figure 1A). Upregulation of GATA-2 might represent genetic compensation phenomenon [48] that occurs as the consequence of perturbation of LSP1 transcriptional activity. However, our comprehension of the molecular mechanisms mediating these genetic compensation events remains poor.

eNOS is a large protein molecule that is dynamically regulated at the transcriptional, post-transcriptional and post-translational levels, including protein–protein interactions [7,49]. We report in this study that the two proteins LSP1 and eNOS are associated with each other and form protein complex in unstimulated ECs. Furthermore, our results of impaired, agonist-stimulated NO release from EA.hy926 cells following depletion of human LSP1, and observation that LSP1 knockout mice have impaired NO-dependent vasodilation provide evidence that the interaction between the two proteins is relevant to the regulation of eNOS function. Other evidence for LSP1 modulation of eNOS activity is the identification of multiple novel phosphorylation sites as well as the absence of conserved inhibitory phosphorylation sites through LC-MS/MS analysis of eNOS purified from ECs transduced with LSP1-HA adenovirus. Our confocal fluorescence microscopy study denotes that colocalization of LSP1 and eNOS takes place primarily in perinuclear region of EA.hy926 cells. Our data that eNOS is predominantly localized in perinuclear region and in discrete areas of plasma membrane are consistent with previous studies [50] that establish the existence of two pools of active eNOS. Remarkably, both native LSP1 and stably expressed tGFP-tagged LSP1 proteins have a similar pattern of colocalization with eNOS in human macrovascular ECs. However, native LSP1 gave rise to a stronger colocalization signal, likely because it has the proper conformation and post-translational modification to complex more avidly with eNOS in ECs. It has been demonstrated that the perinuclear region is the most efficient location for phosphorylation of eNOS and release of vasodilatory NO from active enzyme [50,51,52,53] in ECs. Based on the above investigative work, it was evident that selective localization of LSP1 with eNOS in perinuclear region is plausibly implicated in activation of eNOS to release vasodilatory NO. In this study, no specific fluorescent signal was observed in the negative control slides in which ECs were incubated with the secondary fluorescent probes only without primary anti-LSP1 and anti-eNOS antibodies. Since we have relied on CO-IP and confocal fluorescence imaging to reveal LSP1 interaction with eNOS, we cannot ascertain that the interaction we have observed between the two proteins is direct or indirect, as this would require other protein–protein interaction detection methods, such as a yeast two-hybrid assay. However, there are numerous limitations and drawbacks of those techniques [54,55,56].

Even though there have been extensive studies on post-translational regulation of eNOS, there is a paucity of information available regarding signaling cascades that regulate protein stability of eNOS and its susceptibility to proteolysis. In our data, we have observed truncated 100 kDa eNOS fragment in aortic tissues from LSP1-KO mice in vitro (under denaturing conditions) and in vivo (under non-denaturing conditions). Furthermore, the appearance of 100 kDa eNOS cleavage fragment exclusively in LSP1-KO aortae following immunoprecipitation of eNOS and non-denaturing lysis conditions suggests its expression in vivo and rule out the possibility of acidic hydrolysis or other in vitro cleavage phenomenon as the mechanism for the appearance of the 100 kDa band. Moreover, the preponderance of the 100 kDa eNOS band in the vascular tissues of LSP1 KO mice may imply that LSP1 confers protection to eNOS against the proteolysis of some unknown proteases. Remarkably, the 100 kDa eNOS degradation fragment was detected in our study after probing of immunoblots with two well characterized mouse monoclonal antibodies raised against two distinct epitopes in C-terminus of eNOS, therefore supporting the identity of 100 kDa protein as an eNOS cleavage product and suggesting that it is most likely derived from the C-terminus of eNOS proteins. Our results are in line with previous observations [57,58] revealing that the 100 kDa cleavage product is derived from the C-terminus of eNOS. Furthermore, we have identified the 100 kDa eNOS immunoreactive band in heart tissues from LSP1 KO mice (Figure S3A), and we have observed that eNOS is completely cleaved into the 100 kDa fragment in the mesenteric arteries of LSP1 KO mice (Figure S3B). Apart from the 100 kDa cleavage eNOS protein, we have also observed additional immunoreactive bands at a molecular weight of approximately 75 kDa and 70 kDa in immunoblots from LSP1 KO aortae and mesenteric arteries (Figure 6D) and in vivo (Figure 6B), which represent potential eNOS truncated isoforms. Those bands were strongly reactive with rabbit anti-eNOS polyclonal antibody. Intriguingly, one investigative work has shown that exposure of eNOS to superoxide radical species results in cleavage of the enzyme into the 75 and 60 kDa fragments [59]. Furthermore, the 70 and 60 kDa eNOS truncated forms have been reported in two earlier studies [60,61]. To substantiate whether these immunoreactive bands are derived from eNOS, we immunoprecipitated eNOS from both the WT and LSP1-KO aortae, as bands were more enriched in aortae than in mesenteric arteries (Figure 6D). Two peculiar bands at molecular weights of 75 and 70 kDa were observed in the eNOS immunoprecipitate lane from LSP1-KO aortae (Figure 7A, Lane 2) and were absent in the WT eNOS immunoprecipitate lane. LC-MS/MS analysis has successfully recovered unique eNOS peptides in both bands. These data suggest that LSP1 modulates proteolytic degradation of eNOS. It is plausible to propose that deficiency of LSP1 renders eNOS unstable and predisposes eNOS to proteolytic degradation in vivo. However, the mechanism for preferential truncation of eNOS to 75 and 70 kDa fragments and/or to 100 kDa band fragment remains to be fully elucidated. Remarkably, Coomassie blue staining analysis of eNOS immunoprecipitates suggest that eNOS purified from LSP1 overexpressing ECs lysate was markedly stable and less susceptible to proteolytic degradation compared to eNOS purified from the control ECs transduced with CMV-null adenovirus (Figure 7A, Lanes 3 and 4). Intriguingly, we have observed a potential interaction between human LSP1 and 100 kDa truncated eNOS fragment (Figure 3B). These data advocate the correlation between LSP1 and truncated eNOS proteins and suggest that not only does LSP1 modulate the expression of truncated eNOS isoforms but also that it likely interacts with them in ECs.

It is well established that dimerization of eNOS is crucial for the functionality of the enzyme. It has been demonstrated that N and C terminal eNOS truncation mutants exert dominant negative effect on catalytic activity of eNOS through competition with full-length eNOS monomer and impairment of eNOS dimerization [62]. Furthermore, it has been revealed that a novel, stably expressed eNOS truncated isoform in patients with coronary artery disease has incompetent response to calcium–ionomycin stimulation and has dominant negative effect on NO synthesis from full-length eNOS [63]. Based on the above investigative studies, we postulate that eNOS truncation fragments are potentially implicated in pathogenesis of hypertension associated with LSP1 deficiency. Furthermore, it is plausible to speculate that eNOS truncation fragments contribute to the incompetence of eNOS activity and diminished eNOS-mediated NO release in LSP1-KO mice through inhibition of eNOS dimerization and via a dominant negative effect on NO synthesis from full-length catalytically active eNOS. In our study, although we observed eNOS truncation fragments in vitro and in vivo, we cannot ascertain what intracellular conditions are necessary for the generation of these fragments in vivo. Considering that some eNOS truncation fragments harbor reductase domain of eNOS, which can reduce molecular oxygen and generate superoxide, it remains to be elucidated whether these truncated eNOS proteins could potentially generate reactive oxygen species in vivo, thereby contributing to scavenging eNOS-derived NO and endothelial dysfunction.

To address the cause of hypertension in LSP1-KO mice, we assessed the expression of eNOS in vascular tissues of the WT control mice and LSP1 null mice. LSP1-KO mice have exhibited substantial upregulation of eNOS expression in aortae, and in mesenteric arteries. The discrepancy in eNOS expression between LSP1-depleted ECs and vascular tissues of LSP1 KO mice could be attributed to the complex regulation of eNOS expression in vivo in LSP1 KO mice. Our results are in line with previous studies which demonstrate that there is upregulation of eNOS expression in many hypertension models [64,65,66]. Increased expression of eNOS has also been reported in cardiac tissues of spontaneously hypertensive rats as compensatory effort against high arterial blood pressure [67]. Since endothelial dysfunction in hypertension is associated with low NO bioavailability, upregulation of eNOS expression in LSP1 KO mice is potentially mediated by loss of normal negative feedback of NO on eNOS expression [68,69,70]. The compensatory upregulation of eNOS in setting of hypertension is perceived as futile process since upregulated eNOS is uncoupled dysfunctional enzyme that generates superoxide rather than NO and thereby causes scavenging of NO and aggravates vascular oxidative stress [66,71,72]. Our data demonstrate that upregulated eNOS in LSP1 KO mice is an uncoupled enzyme and is dissociated into 100 kDa truncated eNOS band. Furthermore, eNOS upregulation in LSP1 KO mice could be a compensatory mechanism against high blood pressure in LSP1 KO mice that appears to restore NO bioavailability and to ameliorate for increased cleavage/dissociation of eNOS in vivo in LSP1 KO mice.

Our current study suggests that LSP1 exerts a regulatory effect on eNOS at two distinct transcriptional and post-translational phases. Knockout and overexpression of human LSP1 have revealed the positive regulatory role of LSP1 on eNOS transcription. The nuclear localization of LSP1 in ECs favors its role as a transcriptional coactivator. At post-translational levels, LSP1 exercises its effect on eNOS at multiple aspects. Firstly, LSP1 modulates enzymatic activity of eNOS as manifested by marked reduction in agonist-stimulated NO output following knockdown of human LSP1. Secondly, adequate expression of LSP1 is crucial to preserve dimerization status of eNOS and to halt the dissociation of native eNOS into truncated eNOS isoforms in vivo. Our data suggests that the role of LSP1 in modulating NO output from active eNOS as well as in maintaining proper dimerization status of eNOS is mediated by biochemical interactions between two proteins (eNOS and LSP1) in ECs. Furthermore, it is intriguing to find that eNOS in cardiovascular tissues of LSP1 KO mice is more susceptible to cleavage, suggesting the role of LSP1 in maintaining stability of eNOS monomer against proteolytic truncation. It is remarkable to observe how LSP1 as a cytoskeleton-associated and calcium-binding protein modulates eNOS at multiple complex levels of regulation.

## Data Availability

The data supporting reported results remains intact in the authors’ labs.

## Supplementary Materials

The following supporting information can be downloaded at: https://www.mdpi.com/article/10.3390/biom14010111/s1, Table S1: Selected unique peptides from mouse eNOS (UniProt Accession Number: P70313) identified through tandem mass spectrometry analysis of eNOS immunoprecipitate from aorta lysates of LSP1 KO mice; Figure S1: Validation of CRISPR editing via T7E1 endonuclease assay and locus specific PCR; Figure S2: Western blotting analysis of unanticipated protein level mutations following CRISPR editing of LSP1 gene; Figure S3: Identification of 100 kDa eNOS band in Cardiovascular tissues of LSP1 KO Mice; File S1: Mass Spectrometry Material-1; File S2: Mass Spectrometry Material-2.
