# Supplementary material for: Lymphocyte-Specific Protein 1 Regulates Expression and Stability of Endothelial Nitric Oxide Synthase
Source: Biomolecules. 2024 Jan 15;14(1):111. doi: 10.3390/biom14010111 (PMC10813790; doi:10.3390/biom14010111)
Supplement: Supplementary file 1 [file biomolecules-14-00111-s001.zip › Supplementary figures.pdf]

Supplementary Table S1

Selected unique peptides from mouse eNOS (Uniprot Accession Number: P70313) identified through tandem mass spectrometry analysis of eNOS immunoprecipitate from aorta lysates of LSP1 KO mice. Small letters represent posttranslational modifications. Peptides 1 and 2 represent tryptic peptides from 75kDa band, whereas peptides 3 to 5 are derived from 70 kDa band.

Peptide sequence	Intensity	charge state	Mass over charge m/z
1- EVANAVkISAsLmGTVMAKR	9.90E+05	+3	738.719
2- LSTQAESLQLLPGLTHVHR	2.57E+04	+4	525.8024
3- ESSNTDSAGALGTLR	1.42E+05	+2	739.8625
4- DLCDPHR	1.40E+05	+2	456.7102
5- TYVQDLLR	2.54E+05	+2	504.287

Supplementary Figure S1

A

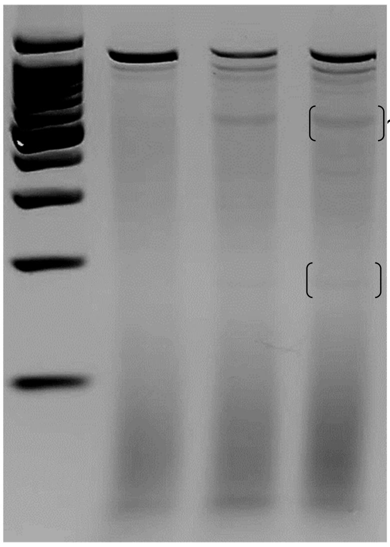

B

Forward primer

CTACACTGATCGAAGAGGCACAGccttaccctcagggagccccaaactga
tgagggaggcatagccctgccctcaggtagccccaggctgatggaggagg
catagccctgctgagcctcaaggagtcctggactgatggaggaagcacag
ttctcaggctgccccagaactgacgggggaggcatagccctgccctcagg
tattctgaccacaggagacacagcccagagccagctctgtggcagacagg
gtctcccacacttggagtccagggcctgagcgggcgccgtgaggtgagtg
tgggccctgggcagaggaggcagcagccgcccaggcctaagctccccct
gctaccctcagcctcagcctgaagccc**tcggaggcccctgaactgg**atga
ggacgagggctttggcgactggtcccagaggccagagcagcggcagcagc
acgagggggcgcagggcgccttggacagcggagagcccccccagtgcagg
agtcctgagggggagcaagaggacaggtgagtgagggcctcgagggcggg
cgctgggcagagcagggctccctctggacctcgagggcgggcgctgggca
gagcagggctccctctggacctcgagggcgggcgctgggcagagcagggc
accgcggagctcccttcaggccctcaacctgcgcctgagcagccacccca
tcctcccctttccaagcagagcctgaaatgcccccatggccaggaaaggg
ggagacaggaggggagggaggaagagggcgagacagcatggtggccggcg
aggggccgcgaggaaacctcattctggggctccccagggcccctgggccc
catccaggcagctgcgaggccaggccgggtgctgccacatcagcacacag
ctgcggccgcttccgtaatggaggctgagagcccgccgccgccccttcct
ccctcggggctggctgatctccattcccacggaggggag**CAGGTATTTCC**
AGATGTGCATG

Reverse primer

C

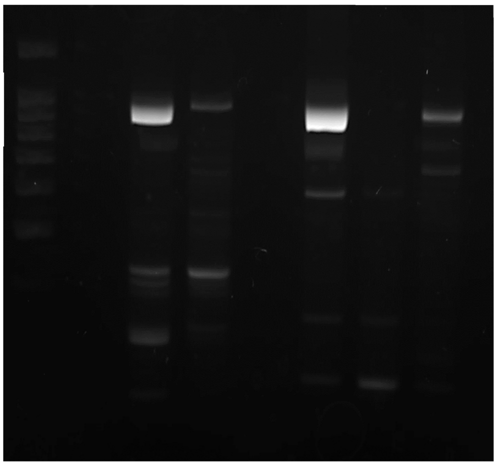

Figure S1. Validation of CRISPR editing via T7E1 endonuclease assay and locus specific PCR. A. genomic PCR amplicons spanning exon 1 of *LSP1* gene were generated from unedited wild-type (WT) control and LSP1 sgRNA-2 KO cells. Reannealing and treatment of PCR products with T7E1 endonuclease yielded two predicted cleavage PCR products of 614 and 377 bp from two different populations of cells transduced with LSP1 sgRNA-2 Cas9 lentiviruses confirming formation of insertions/deletions (indels) and successful editing at sgRNA2 target site. Digestion products were not observed in unedited WT control cells. **B.** The sequences highlighted in bold are the PCR primers, and sequence marked in green is the target region for LSP1 sgRNA-2. **C.** Using PCR-based genotyping protocol, genome edited LSP1 locus via sgRNA1 was PCR amplified utilizing locus specific primers and PCR amplicons were resolved on 4% Agarose gel for 1 hr. WT controls yielded prominent homoduplex DNA bands of predicted size. The presence of PCR amplicons differing from wild type controls indicates mutagenesis in sgRNA target 1. PCR products from Mutant 1 and 3 populations of LSP1 sgRNA-1 KO cells migrate more slowly than unedited controls, suggesting small homozygous insertions. Mutant 2 population of LSP1 sgRNA-1 KO cells exhibited truncated PCR amplicons suggesting formation of large deletions at LSP1 sgRNA1 target site. Asterisks show indels.

Supplementary Figure S2

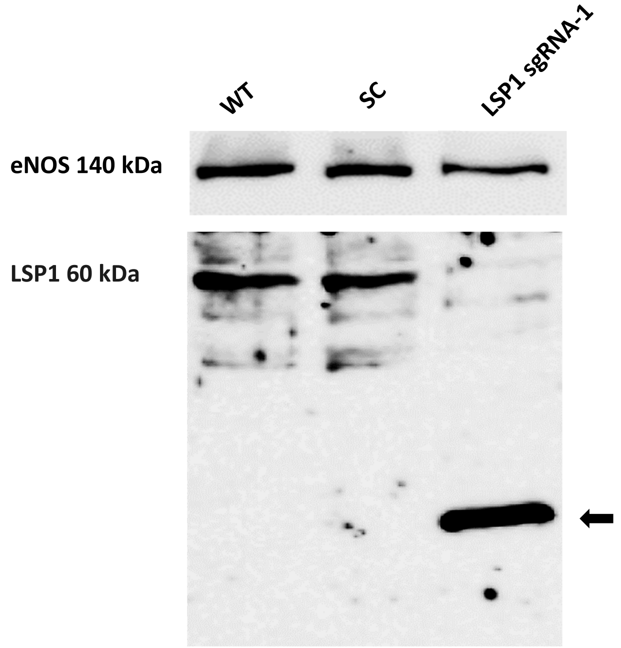

Figure S2: **Western blotting analysis of unanticipated protein level mutations following CRISPR editing of LSP1 gene**. Original uncropped immunoblot of wild type (WT), scramble control (SC), and LSP1 sgRNA-1 KO EC lysates revealed unusual appearance of truncated C-terminal LSP1 protein following CRISPR editing. Truncated LSP1 proteins have been detected by probing immunoblots with rabbit polyclonal anti-LSP1 antibody in two separate experiments. Truncated LSP1 band is absent in WT and SC control lanes. One representative immunoblotting is shown. Black arrow indicates novel truncated protein. Observation of truncated protein confirm CRISPR-induced frameshift mutation in LSP1 gene and successful editing at the protein level.

Supplementary Figure S3

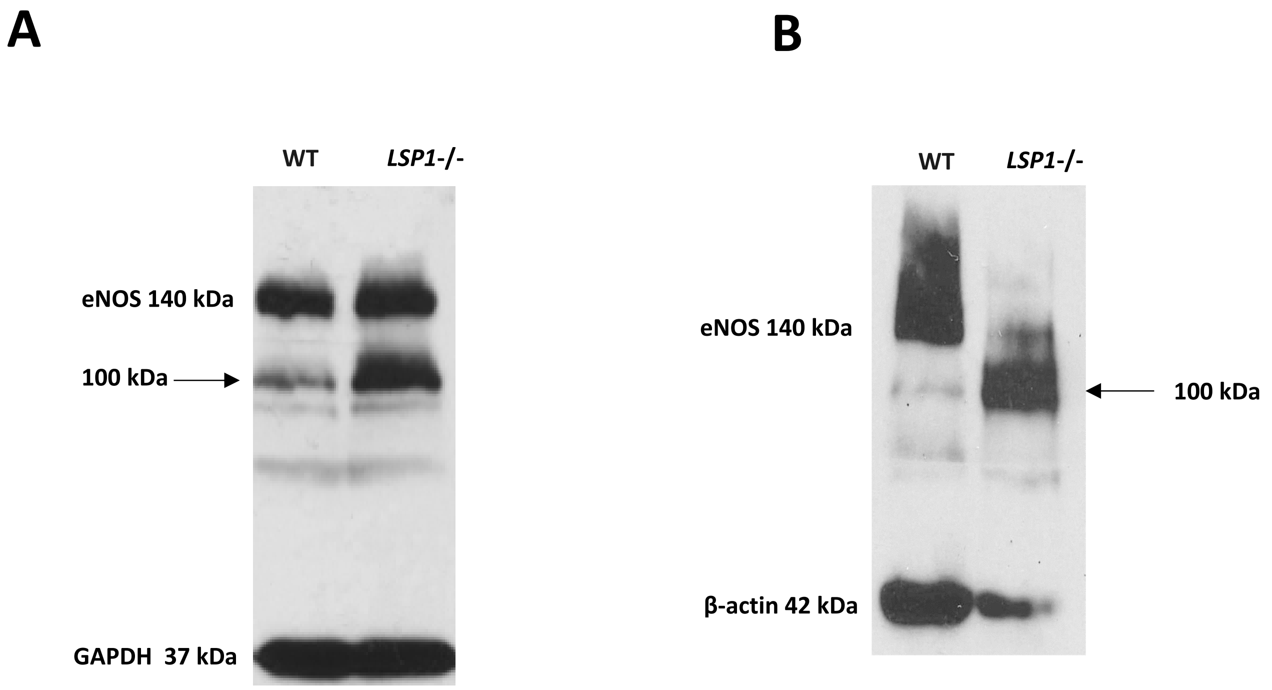

Figure S3: **Identification of 100 kDa eNOS band in Cardiovascular tissues of LSP1 KO Mice**. Heart tissue **(A)** and mesenteric arterial lysates **(B)** from WT and LSP1 KO mice were homogenized by cryogrinding, lysed in ice-cold RIPA buffer, and were subjected to SDS-PAGE and immunoblot analysis. Original uncropped immunoblots are displayed. Arrows point to unique 100kDa eNOS cleavage protein. Blots were probed with C-terminal specific anti-eNOS mouse monoclonal antibody. Representative blots for eNOS 100 kDa fragment from at least three independent experiments are shown. Complete cleavage of eNOS in mesenteric arteries is representative of one experiment where mesenteric arteries from four age-matched WT and LSP1 KO mice were pooled into single protein sample and analyzed by immunoblotting.
